# Functional and Nutraceutical Significance of Amla (*Phyllanthus emblica* L.): A Review

**DOI:** 10.3390/antiox11050816

**Published:** 2022-04-22

**Authors:** Maryam Gul, Zhi-Wei Liu, Iahtisham-Ul-Haq, Roshina Rabail, Fatima Faheem, Noman Walayat, Asad Nawaz, Muhammad Asim Shabbir, Paulo E. S. Munekata, José M. Lorenzo, Rana Muhammad Aadil

**Affiliations:** 1 National Institute of Food Science and Technology, University of Agriculture, Faisalabad 38000, Pakistan; maryamgul1608@gmail.com (M.G.); roshina.rabail@gmail.com (R.R.); fatimafaheem538@gmail.com (F.F.); 2 College of Food Science and Technology, Hunan Agricultural University, Changsha 410128, China; zwliu@hunau.edu.cn; 3 Kauser Abdulla Malik School of Life Sciences, Forman Christian College (A Chartered University), Lahore 54600, Pakistan; iahtisham@fccollege.edu.pk; 4 College of Food Science and Technology, Zhejiang University of Technology, Hangzhou 310014, China; nomanrai66@zjut.edu.cn; 5 Shenzhen Key Laboratory of Marine Microbiome Engineering, Institute for Advanced Study, Shenzhen University, Shenzhen 518060, China; 007298@yzu.edu.cn; 6 Centro Tecnológico de la Carne de Galicia, Avd. Galicia No. 4, Parque Tecnolóxico de Galicia, San Cibrao das Viñas, 32900 Ourense, Spain; jmlorenzo@ceteca.net; 7 Universidade de Vigo, Área de Tecnoloxía dos Alimentos, Facultade de Ciencias, 32004 Ourense, Spain

**Keywords:** polyphenols, ascorbic acid, antioxidant activity, cardiovascular protection, hyperlipidemia, diabetes, health promotion

## Abstract

*Phyllanthus emblica* L. (also popularly known as amla) is a tree native to the India and Southeast Asia regions that produces fruits rich in bioactive compounds that could be explored as part of the increasing interest in naturally occurring compounds with biological activity. Thus, this review aims to highlight the nutritional aspects, rich phytochemistry and health-promoting effects of amla. Scientific evidence indicates that polyphenols are central components in fruits and other sections of the amla tree, as well as vitamin C. The rich composition of polyphenol and vitamin C imparts an important antioxidant activity along with important in vivo effects that include improved antioxidant status and activity of the endogenous antioxidant defense system. Other potential health benefits are the anti-hyperlipidemia and antidiabetic activities as well as the anticancer, anti-inflammatory, digestive tract and neurological protective activities. The promising results provided by the studies about amla bioactive compounds support their potential role in assisting the promotion of health and prevention of diseases.

## 1. Introduction

*Phyllanthus emblica* L. (popular known as amla or Indian gooseberry) is an ephemeral tree belonging to the *Euphorbiaceae* family. Amla fruits are edible and are mainly found in regions of India, Southeast Asia, China, Iran, and Pakistan [1]. Amla has an important role in the traditional medicine of India to reduce anxiety and burning sensation in skin and eyes, improve anemic condition, favor the health of the male reproductive system and reproduction, facilitate digestion, improve liver health, and also exert a tonic effect in the cardiovascular system [2,3].

The fruit of *P. emblica* L. is one of the most popular botanicals, with a wide range of uses in the medicinal, cuisine, and cosmetic industries. This is the first tree to be “produced in the universe”, according to ancient Indian mythology [4]. It is a great nutritional supplement with several medicinal benefits [5]. Due to the abundance of phenolic compounds, Emblic fruit could be regarded as a plant source for natural antioxidants and nutraceuticals or medicinal components. Consumers like Emblic fruit because of its unique flavor and pleasant smell. In various animal and human investigations, amla has been proven to have anti-hyperglycemic, hypoglycemic, anti-inflammatory, anti-hyperlipidemic, and antioxidant activities [1]. Amla is rich in antioxidants such as gallic acid, ascorbic acid and phenolic compounds and thus helps the body’s immune systems and digestion [6]. Thus, due to the increasing interest and the potential of *P. emblica* L., this review aims to provide an overview of the nutritional composition, phytochemistry and potential health benefits associated with the consumption of phytochemicals naturally found in amla.

## 2. Nutritional Composition of Amla

Amla fruits are a relevant source of carbohydrates that account for >70 g/100 g dry weight (DW) (Table 1). Fiber is another relevant component (7.2–16.5 g/100 g DW) as well as contents of protein, minerals such as (iron, calcium and phosphorous), and fat (2.0–4.5, 2.1–3.1, and 0.2–0.6 g/100 g DW, respectively) [7,8,9,10,11,12]. The variability in the composition of amla fruit has been attributed to the cultivar in many studies [10,11,12].

Another important component found in amla fruit is ascorbic acid (vitamin C). Values between 193 and 720 mg/100 g have been reported in different studies that evaluated a different variety of amla [8,9,10,11,12]. Although the optimum recommended daily intake has not been defined yet due to the emergency of new factors from modern society, many governmental health authorities around the globe established Recommended Dietary Allowance (minimum level to meet the need for a healthy person for a day) that varies between 40 and 110 mg vitamin C/day [13]. Moreover, the Australian and China health authorities have proposed a daily intake of 190–220 mg/day. In this sense, a serving portion of at least 100 g of fresh amla fruits (2–3 pieces) from any of the varieties indicated in Table 1 should suffice the daily need for vitamin C. Comparatively, *P. emblica* L. juice can present more vitamin C content than any other fruits such as apple, lime, pomegranate, and some types of grapes [14,15]. Additionally, other nutritionally relevant compounds found in amla fruits are vitamins A, B1, and E (290 IU, 30 mg/100 g, and 0.17 mg/100 g) as well as calcium and iron (25 and 1 mg/100 g) [8].

## 3. Phytochemistry of Amla

**Figure 1:**
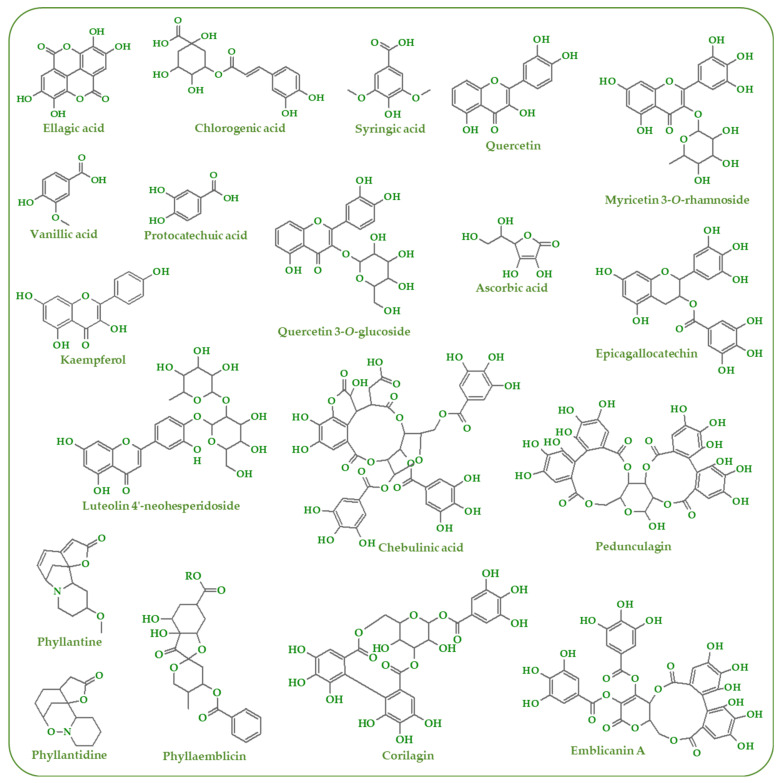
Phytochemicals found in amla.

Amla has been found to possess rich phytochemistry distributed in different sections of the plant (fruits, leaves, and roots). Polyphenols (Figure 1) comprise the main group of secondary metabolites wherein several compounds belonging to phenolic acids, flavonoids, tannins, other phenolics and derivatives compounds have been reported in different studies.

Regarding the phenolic acids, the presence of hydroxybenzoic acids (4-hydroxybenzoic acid, coumaric acid, gallic acid, protocatechuic acid, syringic acid, and vanillic) acid were identified in the fresh fruit and commercial products prepared from the fruits [16,17,18,19,20]. Gallic acid is the only hydroxybenzoic acid reported in leaves and branches [21]. The presence of hydroxycinnamic acids (caffeic acid and chlorogenic acid) was indicated only in amla fruits [16,17,18]. Another class of compounds reported in the amla plant is flavonoids (particularly flavonols, flavones, flavanones, and flavan-3-ols). Flavonols are widely distributed in the different sections of the amla plant. Kampferol their derivatives (dihydrokaempferol, kaempferol 3-b-dglucopyranoside, kaempferol 3-o-rhamnoside, kaempferol-3-o-α-l-(6″-ethyl)-rhamnopyranoside, and kaempferol-3-o-α-l-(6″-methyl)-rhamnopyranoside) are found in fruits, leaves branches and shoots [19,21,22,23]. In a similar way, quercetin and its derivatives (quercetin 3-b-_D_-glucopyranoside, quercetin 3-O-glucoside, quercetin 3-O-rhamnoside, and rutin) are distributed in fruits, leaves and branches [16,17,18,19,21,23].

Regarding flavones, the presence of apigenin, luteolin, and myricetin was indicated in the fresh fruits and fruit commercial products [18,19]. Myricetin 3-O-rhamnoside was reported only in the leaves and branches of the amla tree [21]. Interestingly, flavanones and flavan-3-ols were reported only in leaves and branches [21]. The identified flavanones were eriodictyol, naringenin, and their derivatives ((S)-eriodictyol 7-O-(6″-O-galloyl)-β-D-glucopyranoside, (S)-eriodictyol 7-O-(6″-O-trans-p-coumaroyl)-β-D-glucopyranoside, naringenin 7-O-(6″-O-galloyl)-glucoside, naringenin 7-O-(6″-O-trans-p-coumaroyl)-glucoside, and naringenin 7-O-glucoside). Regarding flavan-3-ols, the detected compounds were epicagallocatechin, epigallocatechin 3-O-gallate, and gallocatechin.

Tannins is another key group of phenolic compounds found in amla fruits, leaves and branches. Many studies indicate the presence of ellagitannins, which includes chebulinic acid, chebulagic acid, corilagin, emblicanin A and B, geraniin, isocorilagin, pedunculagin, phyllanemblinins A–F, and punigluconin [20,23,24,25]. Ellagic acid and their derivatives (decarboxyellagic acid and 3′-O-methylellagic acid 4-O-α-L-rhamnopyranoside) were also reported in fruits, leaves and brunches [21]. Hydrolysable tannins (1,2,3,4,6-penta-O-galloyl-β-_D_-glucose, 1,2,3,6-tetra-O-galloyl-β-_D_-glucose, and 1,2,4,6-tetra-O-galloyl-β-_D_-glucose) and phlorotannins (2-(2-methylbutyryl)phloroglucinol 1-O-(6″-O-β-_D_-apiofuranosyl)-β-_D_-glucopyranoside) are mainly found in leaves and branches of amla [18,21]. The exception is tannic acid, which was reported in amla fruit [21]. Moreover, other phenolics (2,4-di-tert-butylphenol and Phenol, 3,5-bis (1,1-dimethylethyl)) were also reported amla fruit [26]. Additionally, alkaloids (especially phyllantine and phyllantidine) were also reported in amla [7].

## 4. Potential Health Benefits

### 4.1. Antioxidant Activity

Diverse in vitro, in vivo, and human studies support the antioxidant activity of *P. emblica* L. components. In the case of in vitro studies, the content of polyphenols in this fruit has also been associated with high antioxidant activity [20,27,28], particularly with the capacity to scavenge free radicals such as the 1,1-diphenyl, 2-picrylhydrazyl (DPPH) radical [9,12,16,17,18,27,29,30,31,32]. Other studies also reported the antioxidant activity of amla phytochemicals by 2,2′-azino-bis(3-ethylbenzothiazoline-6-sulfonic acid) (ABTS) and NO radical scavenging methods, the Ferric Reducing Antioxidant Power (FRAP) [33], and LDL oxidation assay method [19]. Additionally, amla polyphenols can also scavenge superoxide anion and hydroxyl free radicals as well as chelated iron (III) [20].

The observed antioxidant activity observed from extracts and isolated compounds from amla fruit using chemical methods has also been observed in a more complex biological system that includes cells, animals, and clinical trials (Table 2). In this case, the antioxidant defense system, has an important role in the protection against oxidative damage in vivo. This system is composed of non-enzyme compounds (glutathione; GSH) and enzymes (such as catalase (CAT), GSH reductase, glutathione peroxidase (GPx), and superoxide dismutase (SOD)) [34].

One example of the protective effect of amla fruit bioactive compounds against oxidative damage is the study carried out by Shivananjappa and Joshi [35] in HepG2 cells. These authors observed that total antioxidant capacity (ABTS method) was improved after 4 h of exposure to different concentrations of extract (25, 50 and 100 μg/mL). The peroxidation level was significantly reduced after 8 h of exposure to 50 and 100 μg/mL of extract. Moreover, the antioxidant defense system was induced (GSH, SOD, CAT, GPx, GSH reductase, and GSH S-transferase) after 12–24 h of exposure to 50 and 100 μg/mL of extract. Similar results were reported in a study with RAW 264.7 cells with up to 100 μg/mL of the extract [36]. Particularly for isolated compounds, ethyl gallate was indicated as the most efficient antioxidant (10–50 µM) to reduce oxidative damage in PC12 cells [32].

Another relevant outcome obtained from these studies is the non-significant cytotoxicity of extracts in the range of concentrations (up to 100 μg/mL) where the antioxidant activity and induction of the antioxidant defense system were observed [32,35,36]. It is worth mentioning that a recent experiment indicated that a higher concentration (200 μg/mL) of extract would be required to reduce reactive oxygen species levels and improve their survivability in myoblasts [37].

The protective effect of amla fruit compounds against oxidative damage has also been observed at the animal level. In this case, the daily administration of 500 mg/kg body weight (BW) of amla fruit extract during 28 days induced the activity of GSH, CAT, and SOD in the thymus of mice. [39]. Accordingly, these authors also observed that lipid peroxidation and reactive oxygen species (ROS) levels were reduced. A similar experiment with amla fruit extract revealed a significant reduction in the lipid peroxidation levels, simultaneously decreased the levels of conjugated dienes and CAT levels, and ameliorated the reduction in SOD levels in the liver of mice (500 mg/100 g BW) caused by arsenic [40]. In another study, the amla fruit extract (50–250 µg/mL) induced SOD and CAT activities and reduced lipid peroxidation in the kidneys of mice [41].

Additionally, a study using the extract from amla leaves (200–400 mg/kg BW) indicated a similar protective effect in diabetic mice by reducing inducing the activity of GSH, GPx, SOD, and CAT activity and also reducing lipid peroxidation [38]. However, this experiment did not indicate significant effects on the antioxidant status of healthy animals consuming the amla extract. In another experiment carried out by Reddy et al. [44], the protective role of *P. emblica* L. fruit extract was observed in mice subjected to alcohol-induced oxidative stress. The authors indicated that polyphenols (especially tannins and flavonoids) present in this fruit extract significantly reduced oxidative stress by scavenging NOx.

Amla antioxidants have also been associated with improvements in the antioxidant status in humans. A recent clinical trial with smokers (randomized, double-blind placebo-controlled design) also supports the role of amla fruit as a relevant option of natural antioxidants [42]. In this study, a significant reduction in the peroxidation level and increased antioxidant status were observed in subjects that consumed 250 mg (twice a day) for 60 days. Another clinical trial with subjects diagnosed with metabolic syndrome (randomized, double-blind and placebo-controlled design) indicated that consuming either 250 or 500 mg capsules (twice a day) for 12 weeks reduced the lipid peroxidation levels and induced GSH levels [43]. Conversely, the consumption of 125 mg capsules (4 capsules/day) had non-significant effects on the antioxidant status in healthy subjects (randomized, double-blind, placebo-controlled, and crossover design) [6].

These studies indicate that amla phytochemicals can exert antioxidant activity by limiting the formation of oxidation products, increasing antioxidant status, and also inducing the endogenous antioxidant defense system. Particularly for clinical trials, the effect is promising to prevent oxidative induced by lifestyle (smoking) or the management of diseases (metabolic syndrome).

The polyphenols naturally present in amla also exert other biological effects beyond antioxidant activity. Table 3 indicates the phenolic compounds, individually or collectively, associated with biological effects. These studies indicate amla fruit as the most studied source of bioactive compounds (especially polyphenols from different groups indicated in Section 3). In most cases, the biological effect is attributed to more than one polyphenol composing the experimental sample. The biological effects of amla polyphenols are discussed in the following sections.

### 4.2. Cardioprotective Activity

Hyperlipidemia is one of the major causes of cardiovascular disorders [68], but amla bioactive compounds may assist in the management of this condition. Different studies have been shown the protective effects of amla and/or its constituents against cardiovascular diseases. One example is the study conducted by Nambiar and Shetty [19] who studied the effect of amla juice (myricetin, gallic acid, and kaempferol as main polyphenols) on low-density lipoprotein (LDL) oxidation. According to the authors, limited the uptake of LDL oxidation in macrophages and LDL cholesterol oxidation was reduced by 90%. Another study indicated that amla polyphenols (emblicanin A and B, punigluconin, and pedunculagin) limited fibrosis formation in cardiovascular tissue of mice subjected to schemia and reperfusion [51].

Madan et al. [69] tested the effect of amla supplementation in beetal kids and observed reductions in the levels of LDL, cholesterol, and blood glucose to the non-supplemented group. Another experiment showed that hydroalcoholic amla extract reduced the arterial mean blood pressure, and serum sodium levels and aided to increase the potassium levels in deoxy-corticosterone acetate salt-induced hypertensive mice [52]. This study also indicated that *P. emblica* L. regulated the endogenous antioxidant system, eNOS, activation of serum nitric oxide (NO), and serum electrolytes level.

**Figure 2:**
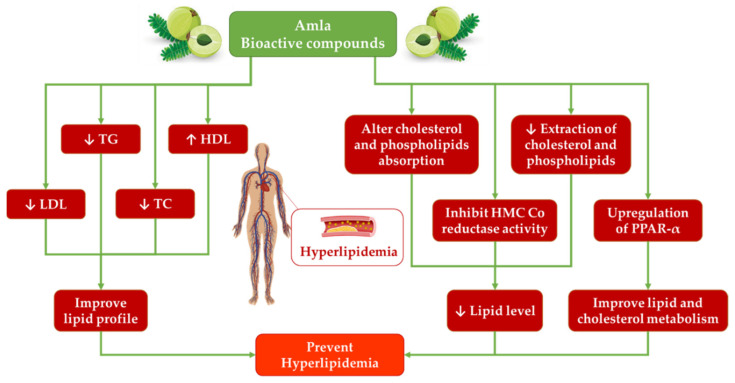
Anti-hyperlipidemic role of amla.

The polyphenol-rich extracts of *P. emblica* L. reduced metabolic changes caused by excessive fructose consumption (alteration of triglyceride total cholesterol levels and sterol regulatory element-binding protein 1 (SREBP-1) expression) in an animal model. *P. emblica* L. (containing gallic acid, chebulagic acid, geraniin, ellagic acid, and corilagin) reduced and even inhibited the enhanced mitochondrial COX-2, MDA, and Bax expressions in the liver and regulated Bcl-2 expression, but peroxisome proliferator-activated receptors-α (PPARα) and SREBP-2 expressions were unaffected [55]. In another experiment, polyphenol-rich *P. emblica* L. extract also increased PPARα protein (involved in the regulation of cholesterol and lipid metabolism) expression and decreased cholesterol levels in mice [66]. Similarly, a related study carried out with mice that consumed amla juice (2 mL/kg/day; rich in gallic acid) indicated the activation of PPARα and carnitine palmitoyl transferase (involved in lipid oxidation) [45]. Another interesting outcome of this study was the reduction in the activity of liver enzymes involved in lipogenesis (malic enzyme, fatty acid synthase, and glucose-6-phosphate dehydrogenase). However, another study with mice in a high-fat diet (30%), indicated that *P. emblica* L. ethanolic extract reduced the serum triglycerides but no effects were observed in LDL, very-low-density lipoprotein (VLDL), or high-density lipoprotein (HDL) serum levels [70]. The antihyperlipidemic and cardioprotective potential of amla is represented in Figure 2.

At the human level, a 500 mg dose of *P. emblica* L. extract (twice a day) for three months reduced the high sensitive C-reactive protein (CRP), total cholesterol, and LDL levels in Class I obese subjects [53]. Gopa et al. [71] studied the effect of amla fruit capsules (500 mg/capsule; once a day for 42 days) and reported significant reductions in subjects with hyperlipidemia. According to these authors, significant reductions in total cholesterol, LDL, and VLDL levels along with a significant increase in the serum levels of HDL at the end of the trial period were also observed. Moreover, different studies indicated that *P. emblica* L. fruit and extract reduced the VLDL, cholesterol, and LDL levels in hyperlipidemic patients and healthy persons. When administered for 2–6 months, the extract decreased the level of important inflammatory marker CRP, resulting in an enhanced level of HDL and protection against atherosclerosis [71,72].

### 4.3. Antidiabetic Activity

The compounds naturally found in *P. emblica* L. have been associated with protective effects against diabetes. An in vitro study indicated that the activity of the main phytochemicals found in amla (such as ellagic acid and ascorbic acid) reduced the activity of key enzymes involved in glucose digestion (especially amylase and glucosidase) [73].

The protective effects against diabetes have also been reported at the animal level. For instance, a freeze-dried *P. emblica* L. aqueous extract (1.25 g/kg) reduced the levels of serum glucose and triglyceride in diabetic long Evan male mice (induced with streptozotocin) [74]. Likewise, Patel and Goyal [75] observed the antidiabetic potential of *P. emblica* L. juice (1 mL/kg/day) due to the attenuated increase in serum glucose levels of animals with induced diabetes. In another experiment with diabetic mice, the doses of 250 and 500 mg *P. emblica* L. extract/kg (rich in ellagic acid) caused significant reductions in the serum glucose levels and improved the serum insulin levels [46]. Likewise, the experiment carried out by Nain et al. [38] reported a similar outcome from the phytochemical extracted from leaves of *P. emblica* L. According to these authors, the increase in serum insulin and the related reduction in serum glucose were observed for diabetic mice in daily doses between 100 and 400 mg/kg.

Clinical trials also support the health benefits of amla phytochemicals for diabetic patients. For instance, daily doses of up to 3 g of *P. emblica* L. powder extract reduced blood glucose levels in diabetic patients after 21 days of the trial [76]. A similar outcome was reported by Walia et al. [1] who observed significant reductions in blood glucose in diabetic patients after consuming 10 g of amla powder once a day for 90 days. Additionally, flavonoid-rich *P. emblica* L. extract also reduced the risk of neuropathy [60] in diabetic patients. Amla bioactive compounds seem to play a key role in the management of diabetes, particularly in assisting in the restoration of glucose and insulin levels.

### 4.4. Anticancer Activity

Plant-derived polyphenols have been found to improve the protection against cancer in a variety of nonclinical and clinical investigations [77,78]. Specifically, polyphenols inhibit oxidative stress, produce pro-inflammatory chemicals, prevent DNA damage, and increase apoptosis through various mechanisms [79]. Particularly for amla extracts, DNA fragmentation, increased activity of caspase-3, 7, and 8, and up-regulation of Fas protein were observed in the HeLa cell line, indicating activation of the death receptor pathway for apoptosis, whereas caspase-9 remained unaltered [80]. This study also indicated that *P. emblica* L. decreased the invasiveness of MDA-MB-231 cells (in vitro Matrigel invasion study), and no cytotoxicity was seen in normal lung fibroblasts (MRC5). Likewise, pyrogallol (a polyphenol found in *P. emblica* L.) was investigated in human lung cancer cell lines H441 and H520. Pyrogallol’s anti-proliferative effect was achieved via cell arrest in the G2/M phase, which was caused by a drop in cyclin B1, cdc25c, and Bcl-2, as well as an increase in Bax expression [47].

Zhu et al. [81] conducted a study in HeLa cell lines using polyphenolic extract of *P. emblica* L. The extract was capable of inhibiting HeLa cell proliferation by stopping cells in the G2/M phase and promoting apoptosis by inducing apoptotic markers Fas, FasL, and cleaved caspase-8. Huang and Zhong [67] found that gallic acid isolated from *P. emblica* L. leaves induced apoptosis in a hepatocellular cancer cell line (BEL-7404). According to this study, the activity of *P. emblica* L. may be related to the inhibition of the cell cycle in the G2/M phase. Overexpression of Bax and downregulation of Bcl-2 causes a reduction in mitochondrial membrane potential, which activates caspases, resulting in cell death via the apoptotic death-receptor pathway. Additionally, it has also been suggested that *P. emblica* L. play a protective role in chemo- and radiotherapy [48,80,82]. In this sense, the protective effect of amla bioactive compounds seems to have a relevant limiting effect on the progression of cancer progression in different cell lines. However, the evidence supporting the anticancer activity of amla polyphenols is limited and more efforts are still necessary to clarify the mechanisms involved and explored the effects at the animal level and expand the current knowledge.

### 4.5. Anti-Inflammatory Activity

Amla phytochemistry seems to promote a beneficial effect in the context of inflammation, but current evidence is limited. An example of the anti-inflammatory activity in cell model is the study carried out by Li et al. [36]. According to these authors, RAW 264.7 cells treated with amla extract (rich in gallic acid, corilagin, and ellagic acid) displayed lower levels of inflammatory markers (NO release and production of tumor necrosis factor (TNF-α), interleukin-1β (IL-1β), and interleukin-6 (IL-6)) when an inflammatory response was caused by exposure to lipopolysaccharides.

This modulation of inflammatory markers was also observed at an animal level in a study with arsenic exposure [83]. Animals treated with amla extract (500 mg/kg) showed significantly lower serum levels of TNF-α, IL-1β, and IL-6 than animals exposed only to arsenic. Another relevant outcome obtained from *P. emblica* L. extract was the reduction in edema size in mice‘s paws. The natural extract induced the production and release of pain and inflammatory mediators. This effect is suggested to be mediated in a similar way to nonsteroidal anti-inflammatory drugs rather than steroidal medicament way [49,56,84]. In another study with mice, Goel et al. [85] demonstrated the analgesic effects and substantial decrease in abdominal writhing of *P. emblica* L. extract with a dose of 600 mg/kg.

In the case of studies involving humans, a randomized crossover clinical trial with type-2 diabetes subjects indicated that *P. emblica* L. fruit extract (500 mg/day; containing punigluconin, emblicanin-A, emblicanin-B, and peduculagin) decreased platelet aggregation in both single and repeated dosage regimens [24].

### 4.6. Digestive Tract Protection

*P. emblica* L. polyphenols have also been indicated to protect gastrointestinal organs. One of the potential effects of amla bioactive compounds is the potential inhibition of clarithromycin-resistant *Helicobacter pylori* strains in vitro, since this microorganism is a known cause of gastric ulcers [61].

Relevant outcomes were also reported in studies with animals. Al-Rehaily et al. [62] studied anti-secretory and antiulcer activities of *P. emblica* L. extract in mice with different methods to induce gastrointestinal ulcers: ligating pylorus, administrating indomethacin and necrotizing agents (25% NaCl, 0.2 M NaOH, and 80% ethanol), and inducing hypothermia. Both doses (250 and 500 mg/kg) reduced gastric secretion, ulcer index (pylorus-ligated and necrotizing agent-intoxicated ulcer methods), intraluminal bleeding and gastric lesions (hypothermic restraint-induced ulcer method). Particularly for the indomethacin-induced ulcer method, only the animals in the treatment with 500 mg/kg had a significantly lower ulcer index than animals in the control group (treated only with indomethacin).

The potential of amla phytochemicals to protect the liver was reported in the study carried out by Huang et al. [63] with mice with high fat diet-induced non-alcoholic fatty liver disease. According to the authors, the liver of animals treated with amla extract had significant improvements in adiponectin activity and expression of PPAR-α, which improved steatosis. In another experiment, the use of *P. emblica* L. (200 mg/100 g; rich in tannins and gallic acid) in _L_-arginine-induced pancreatitis in mice decreased lipase and IL-10 blood concentrations [59]. This study also revealed that animals in the amla group had more suitable nucleic acid content material, pancreatic protein, rate of DNA synthesis, and pancreatic amylase levels and the histological examination indicated an extensively higher share of smooth cells and a lower inflammatory score. Likewise, the methanolic extract of *P. emblica* L. fruit (100 and 200 mg/kg) reduced the histological alterations in the colon of mice from acetic acid-induced colitis [86].

Another interesting potential health benefit from amla consumption was reported in patients with gastroesophageal reflux disease [64]. The daily consumption of amla extract (500 mg/tablet, twice a day) reduced the severity and frequency of regurgitation and heartburn in comparison to placebo group. The aforementioned experiments support the protection of organs involved in digestion with amla phytochemicals (especially polyphenols) consumption. Moreover, seems reasonable to indicate that modern medicine provides partial support to traditional medicine practices with amla.

### 4.7. Neurological Protection

One of the potential protective effects associated with amla bioactive compound is the attenuation of neurological alterations, particularly the biochemical changes observed in carriers of Alzheimer’s disease. For instance, the administration of amla fruit extract (100 mg/kg; rich in emblicanin A and B) for 60 days in mice reduced the neurotoxicity induced by aluminum chloride [50]. Specifically, significant improvement against the triggering of apoptotic mechanisms (involving apoptotic protease activating factor 1, Bax, and cytosolic cyto c proteins) with reduced acetylcholinesterase activity in the cerebellum was observed in the group treated with amla extract. A related experiment indicated a similar outcome in the expression of Bax, caspases-3 and -9, cytochrome c proteins and also indicated the reduction in tau hyperphosphorylation [54]. Moreover, this study also revealed the involvement of GSK-3β/Akt signaling pathway in the reduced phosphorylation of tau protein in animals treated with amla extract (100 mg/kg for 60 days).

Another relevant outcome reported from the administration of amla extract (up to 200 mg/kg; containing emblicanin A and B, punigluconin, pedunculagin, rutin, and gallic acid) in animals with chemically induced neurological impairment is the improvement of memory and learning deficit in different studies [57,58]. The enhancement of neurological functions associated with amla was also observed in healthy animals [87]. In this case, the improvements were observed in animals that consumed extracts from unripe fruits at 100 and 200 mg/kg doses and ripe fruits at 200 mg/kg, which suggests that neuroactive compounds may be found in unripe rather than ripe amla fruits. It is also relevant to mention that the study carried out by Dhingra et al. [65] indicated some antidepressant possible mechanisms of action associated with amla polyphenols. These authors observed partial inhibition of the antidepressant effect of amla extract was observed in animals co-administered with γ-aminobutyric acid, alpha 1-adrenoceptor, and selective D2-receptor antagonists as well as tryptophan hydroxylase inhibitor. The studies reporting the neuroprotective effects of amla phytochemical support the potential role as assisting agent to attenuate biochemical and physiological changes associated with neurological disorders.

## 5. Conclusions

The rich phytochemistry composition of amla can be seen as a relevant source of compounds with potential health benefits. The antioxidant (from the rich polyphenol composition) is a major property with scientific evidence supporting the direct inhibition of oxidative reactions and the induction of an endogenous antioxidant defense system. Beyond antioxidant activity, seems reasonable to consider with the current level of evidence that amla components (mainly polyphenols) may have a role as supporting source of active compounds to promote health (such as improving antioxidant status in smokers and improving digestive tract protection against stressing agents) and increase the protection against the development of diseases (assisting in the regulation of serum glucose and insulin levels, for instance).

Although a promising scenario can be seen for amla, it is important to promote the progression of studies to strengthen the current evidence with more studies (especially at animal and human levels). Clarifying aspects related to bioaccessibility of bioactive compounds, interaction with gut microbiota, and also exploring technologies and strategies to promote the incorporation into food products (functional foods) are relevant aspects to be explored in future studies.

## Figures and Tables

**Table 1:** Nutritional constituents of amla fruit from different studies.

Variety	Moisture	Carbohydrate	Fiber	Minerals	Protein	Fat	Vitamin C	Ref.
Local variety (no name)	81 g/100 g	14 g/100 g	3.2 g/100 g	0.3 g/100 g	1 g/100 g	0.5 g/100 g	720 mg/100 g	[8]
Local variety (no name)	82.8 g/100 g	7.6 g/100 g	5.1 g/100 g	2.3 g/100 g	2.0 g/100 g	0.3 g/100 g	573 mg/100 g	[9]
NA-7 NA-9 NA-10 Balwant Chakaiya Hathijhool	84.9–87.5 g/100 g	77.2–81.9 g/100 g DW	11.7–16.0 g/100 g DW	2.1–3.0 g/100 g DW	3.0–4.5 g/100 g DW	0.2–0.5 g/100 g DW	489.9–585.0 mg/100 g	[10]
NA-7, Banarasi, Kanchan, Chakaiya and Desi	81.3–84.6 g/100 g	73.8–87.1 g/100 g DW	7.2–22.4 g/100 g DW	2.2 to 3.1 g/100 g DW	2.0 to 3.2 g/100 g DW	0.4–0.5 g/100 g DW	193–315 mg/100 g	[11]
Krishna, Kanchan, NA-7, Chakaiya	85.6–87.7 g/100 g	70.7–73.8 g/100 g DW	13.9–16.5 g/100 g DW	2.3–2.8 g/100 g DW	2.9–3.6 g/100 g DW	0.5–0.6 g/100 g DW	421–506 mg/100 g	[12]

DW: dry weight.

**Table 2:** Antioxidant effect and induction of endogenous antioxidant defense mechanism.

Source	Type of Study	Study Characteristics	Main Outcomes	Ref.
Fruit	In vitro (cell)	PC12 cells; dosage (10–50 µM); and incubation (2 h)	No toxicity; ethyl gallate was the most efficient antioxidant (10–50 µM)	[32]
Fruit	In vitro (cell)	HepG2 cells; dosage (5, 10, 20, 50, and 100 μg/mL); and incubation (4, 8, 12, 16, 20, and 24 h)	No Cytotoxicity (up to 100 μg/mL); reduced lipid hydroperoxides reactive oxygen species levels (50 and 100 μg/mL after 8 h); and increased GSH, total antioxidant capacity, SOD, CAT, GPx, GSH reductase, and GSH S-transferase (50 and 100 μg/mL after 12–24 h)	[35]
Fruit	In vitro (cell)	RAW 264.7 cells; dosage (25, 50, or 100 μg/mL); and incubation (24 h)	No Cytotoxicity (100 μg/mL); increased GSH and SOD activity when challenges with H_2_O_2_ (50 and 100 μg/mL); and reduced MDA level (100 μg/mL)	[36]
Fruit	In vitro (cell)	C2C12 myoblasts; dosage (100 and 200 µg/mL); and incubation (48 h)	Increased cell survivability (200 µg/mL) and reduced ROS levels with increased oxygen consumption (200 µg/mL)	[37]
Leaves	Animal (mice)	Diabetic wistar mice; 100–400 mg/kg BW; oral administration; and 45 days	Induced GSH, GPx, SOD, and CAT activity (200–400 mg/kg BW) and reduced lipid peroxidation (200–400 mg/kg BW)	[38]
Fruit	Animal (mice thymus)	Balb/c male mice; 500 mg/kg BW; oral administration; and 28 days	Improved cell viability, GSH, CAT, and SOD levels and reduced lipid peroxidation, ROS level	[39]
Fruit	Animal (mice liver)	Wistar mice; 5000 mg/kg BW; oral administration; and 24 days	Reduce lipid peroxidation; preserved CD, CAT, and NPSH; and ameliorated SOD reduction	[40]
Fruits	Animal (mice kidney)	Healthy wistar mice; dosage (50, 100, 150, 200, and 250 µg/mL); single application	Increased SOD and CAT (50–250 µg/mL); reduced lipid peroxidation (50–250 µg/mL); and no effect in GSH	[41]
Commercial supplement	Clinical trial	Male smoker subjects (20–60 y); randomized, double-blind placebo-controlled design; 250 mg (twice a day); and 60 days	Increased antioxidant status (FRAP assay) and reduced lipid peroxidation level	[42]
Commercial supplement	Clinical trial	Female and male subjects with metabolic syndrome (30–68 y); randomized, double-blind, and placebo-controlled; 250 and 500 mg per capsule (twice a day); and 12 weeks	Increased GSH level and reduced lipid peroxidation level	[43]
Commercial supplement	Clinical trial	Female and male healthy subjects (36–67 y); randomized, double-blind, placebo-controlled, and crossover; 125 mg per capsule (4 capsules/day)	A non-significant reduction in lipid peroxidation level	[6]

BW: body weight; CAT: Catalase; CAT: Catalase; CD: conjugated dienes; GPx: Glutathione peroxidase; GSH: Glutathione; NPSH: non-protein soluble thiol; ROS: Reactive oxygen species; and SOD: Superoxide dismutase.

**Table 3:** Amla polyphenols and their biological effects beyond antioxidant activity.

Source	Main Active Compounds	Biological Effect	Ref.
Fruit	Gallic acid	Cardioprotective activity	[45]
Fruit	Ellagic acid	Antidiabetic activity	[46]
Fruit	Pyrogallol	Anticancer activity	[47]
Fruit	Emblicanin A and B	Anticancer activity	[48]
Fruit	Emblicanin A and B	Anti-inflammatory activity	[49]
Fruit	Emblicanin A and B	Neuroprotective activity	[50]
Fruit	Myricetin, gallic acid, and kaempferol	Cardioprotective activity	[19]
Fruit	Gallic acid, corilagin, and ellagic acid	Anti-inflammatory activity	[36]
Fruit	Emblicanin A and B, punigluconin, and pedunculagin	Cardioprotective activity	[51,52,53]
Fruit	Emblicanin A and B, punigluconin, and pedunculagin	Anti-inflammatory activity	[24]
Fruit	Emblicanin A and B, punigluconin and pedunculagin	Neuroprotective activity	[54]
Fruit	Gallic acid, chebulagic acid, geraniin, ellagic acid, and corilagin	Cardioprotective activity	[55]
Fruit	Quercetin, rutin, gallic acid, mucic acid, and beta-glucogallin	Anti-inflammatory activity	[56]
Fruit	Emblicanin A and B, punigluconin, pedunculagin, rutin, and gallic acid	Neuroprotective activity	[57,58]
Fruit	Tannins and gallic acid	Gastrointestinal protective activity	[59]
Fruit	Flavonoids	Antidiabetic activity	[60]
Fruit	Polyphenols	Gastrointestinal protective activity	[61,62,63,64]
Fruit	Polyphenols	Neuroprotective activity	[65]
Fruit	Polyphenols	Cardioprotective activity	[66]
Leaves	Gallic acid	Anticancer activity	[67]
